# Characterization of Regional Poison Center Utilization Through Geospatial Mapping

**DOI:** 10.5811/westjem.2020.7.46385

**Published:** 2020-10-20

**Authors:** Travis D. Olives, Bjorn Westgard, Lila W. Steinberg, Jon B. Cole

**Affiliations:** * Minnesota Poison Control System, Minneapolis, Minnesota; † Hennepin Healthcare, Department of Emergency Medicine, Minneapolis, Minnesota; ‡ University of Minnesota Medical School, Department of Emergency Medicine, Minneapolis, Minnesota; § Regions Hospital, Department of Emergency Medicine, St. Paul, Minnesota

## Abstract

**Introduction:**

Penetrance is the annual rate of human exposure calls per 1000 persons, a measure that historically describes poison center (PC) utilization. Penetrance varies by sociodemographic characteristics and by geography. Our goal in this study was to characterize the geospatial distribution of PC calls and describe the contribution of geospatial mapping to the understanding of PC utilization.

**Methods:**

This was a single-center, retrospective study of closed, human, non-healthcare facility exposure calls to a regional PC over a five-year period. Exposure substance, gender, age, and zone improvement plan (ZIP) Code were geocoded to 2010 US Census data (household income, educational attainment, age, primary language) and spatially apportioned to US census tracts, and then analyzed with linear regression. Penetrance was geospatially mapped and qualitatively analyzed.

**Results:**

From a total of 304,458 exposure calls during the study period, we identified 168,630 non-healthcare exposure calls. Of those records, 159,794 included ZIP Codes. After exclusions, we analyzed 156,805 records. Penetrance ranged from 0.081 – 38.47 calls/1000 population/year (median 5.74 calls/1000 persons/year). Regression revealed positive associations between >eighth-grade educational attainment (β = 5.05, p = 0.008), non-Hispanic Black (β = 1.18, p = 0.032) and American Indian (β = 3.10, p = 0.000) populations, suggesting that regions with higher proportions of these groups would display greater PC penetrance. Variability explained by regression modelling was low (R2 = 0.054), as anticipated. Geospatial mapping identified previously undocumented penetrance variability that was not evident in regression modeling.

**Conclusion:**

PC calls vary substantially across sociodemographic strata. Higher proportions of non-Hispanic Black or American Indian residents and >eighth-grade educational attainment were associated with higher PC call penetrance. Geospatial mapping identified novel variations in penetrance that were not identified by regression modelling. Coupled with sociodemographic correlates, geospatial mapping may reveal disparities in PC access, identifying communities at which PC resources may be appropriately directed. Although the use of penetrance to describe PC utilization has fallen away, it may yet provide an important measure of disparity in healthcare access when coupled with geospatial mapping.

## INTRODUCTION

Poison centers (PC) accredited by the American Association of Poison Control Centers (AAPCC) offer high-quality information to callers seeking information and medical consultations for poisoned patients. They serve critical roles in real-time epidemiological surveillance of poison exposures and disease epidemics, and are a key component of our national health surveillance system.^1^ Increased PC utilization has been associated with decreased emergency medical services utilization and unnecessary hospitalizations^2^ and with shortened hospital stays following exposure.^3^,^4^

PC utilization has historically been assessed in terms of *penetrance*, defined as the annual number of calls per 1000 persons in a defined call area.^5^,^6^ Penetrance rises with changes in United States Census Bureau (USCB) population estimates and live birth rates,^7^ suggesting a correlation between population growth and childhood poison exposures. Low PC penetrance is associated with increased healthcare utilization, particularly in children.^5^,^8^ Variations in penetrance have been attributed to seasonality,^6^ changing pediatric population proportions,^9^ limited awareness of PC services,^10^ and suspicion regarding PC cost and safety of personal information.^11^ Social determinants of PC penetrance are less well-defined, although several racial (Black and Native American) and linguistic (low English proficiency and native Spanish-speaking) characteristics are associated with lower PC utilization when compared to White and English-speaking populations.^5^,^10^,^12^,^13^

The use of penetrance has been disputed over time, largely due to a perceived limited efficacy in assessing both PC efforts and outcomes.^9^ Although the AAPCC discontinued its use of penetrance as one of multiple methods to ascribe efficacy to individual PC outreach and promotion efforts in 2001, it was done prior to the advent of easily accessible, geospatial mapping tools to provide a more refined data than at a county level, suggesting that penetrance may once again serve a purpose in identifying areas in which PCs are underused. The same variation in penetrance attributed to sociodemographic variables that led to its discontinuation as a metric for PC accreditation is suggestive of its value in further exploring predictors of PC utilization.

Few studies describe geographic penetrance at a level more granular than county-wide, despite intra-county variability in race, income, ethnicity, and other socioeconomic determinants of health.^14^–^17^ Focal exposure clusters may be localized within close proximity and overlooked within county-wide analyses.^18^ Therefore, exposure patterns may be better understood when mapped geospatially. We hypothesized that highly granular geospatial mapping would reveal previously unidentified sociodemographic predictors of penetrance. The goal of the study was to characterize PC call penetrance by USCB tracts to better characterize variation across the PC catchment area.

## METHODS

### Setting

This was a retrospective study characterizing the group-level demographic characteristics and geospatial distribution of human exposure calls to a regional PC from locations other than healthcare facilities over a five-year period, from January 1, 2010–December 31, 2014. The Minnesota Poison Control System covers a catchment area of nearly 87,000 square miles (greater than 218,000 square kilometers) and serves approximately 5.5 million people. It receives more than 50,000 calls annually; a majority of these originate in sites other than healthcare facilities.

**Population Health Research Capsule**

What do we already know about this issue?

*Poison centers (PC) serve large populations, but call penetrance may vary*.

What was the research question?

*We sought to characterize the geospatial distribution of calls to a single regional PC*.

What was the major finding of the study?

*Calls to a PC vary substantially by sociodemographic strata, with significant geospatial variation in call origin, while regression modelling suggested greater penetrance in regions with higher proportions of non-Hispanic Black and American Indian populations, and >8th grade educational attainment, however variability explained by the model was low*.

How does this improve population health?

*Statistical analyses describe patterns to regional PC callers, but spatially mapping call density may identify areas of low call penetrance to guide outreach efforts*.

Minnesota is a diverse state. Smaller proportions of the population than the national average live in poverty (11.5% vs 15.4%) and fewer report non-English language use (11.5% vs 15.4%), but racial disparities are profound: higher proportions of Blacks and Native Americans in our state live in poverty than nationally (36.5% vs 27.3% and 36.0% vs 28.8% in 2014, respectively).^19^,^20^ Attainment of a bachelor’s degree or higher varies substantially across racial groups, from 8% among Ojibwe to 85% among Asian-Indian residents.^21^ Additionally, the state is home to the country’s largest Somali population, second largest Hmong population, third largest Lao population, and fifth largest Burmese population.^22^ One in seven Liberians in the US resides in Minnesota, while one in 12 of Ethiopian descent resides here.^23^ Overall, 4.25% of our service population possesses limited English proficiency.^24^ Data characterizing statewide health literacy is limited, but suggest that up to one in five patients seeking emergency services possesses limited health literacy.^25^,^26^

Recent multi-patient toxicological exposures in minority communities^27^,^28^ have highlighted the importance of PC penetrance in historically underserved populations. These outbreaks have been concentrated in small geographic areas incompletely captured by county-level geospatial mapping, suggesting a potential benefit to improved understanding of the spatial distribution of PC calls. Despite known multi-patient exposures in language- and ethnic-minority communities, telephonic interpretive services are engaged on average fewer than five times per month in the PC, or less than 0.2% of all calls. While it is plausible that linguistically under-represented and economically disadvantaged segments of the population experience fewer poisonings than others, such disparities raise suspicion for a lack of access to PC services.

Thus, following approval from the governing institutional review board, we queried the National Poison Data System (NPDS) for all closed human-exposure calls originating within Minnesota (Caller site/Exposure site: Own residence, Other Residence, Workplace, School, Restaurant / food service, Public area, Unknown, NULL). The NPDS maintains all call data generated by the nation’s 55 PCs, with nearly continuous real-time database updates.^1^ Because the goal of this study was to characterize non-healthcare penetrance across an entire PC call area, we excluded calls coded as originating from healthcare facilities or referencing exposures occurring in healthcare facilities, as their inclusion would have over-represented a small number of census tracts rather than accurately describe exposure distribution. Calls originating outside of Minnesota, calls without zone improvement plan (ZIP) Codes, and calls for which ZIP Code geocoding was not possible were excluded from analysis. No further exclusions were made.

### Data Analysis

Patient-level data including ZIP code, gender, age, exposure reason (intentional or unintentional), caller and exposure sites were electronically abstracted from NPDS. We then imported call records to Microsoft Excel 2013 (Microsoft Corporation, Redmond, WA). PC data, including postal ZIP Code, were geocoded to USCB tract data (2010) for household income, educational attainment, age, ethnicity and primary language. The resulting dataset was spatially apportioned to USCB tracts based on quantifiable spatial and population overlaps. ArcGIS 10.5 (Esri, Redlands CA) was then used to generate heat maps defining the penetrance of callers to the PC over USCB tracts overlapping 87 Minnesota counties.

We developed a multiple regression model of penetrance using clinically important variables within the USCB dataset, including the continuous (0 to 1.0) proportions of households reporting greater than eighth-grade educational attainment, population <5 years of age, households below the federal poverty line, and households that reported speaking a language other than English. The proportion of the population identifying as Hispanic, non-Hispanic Black, non-Hispanic American Indian, and non-Hispanic Asian were also included. We evaluated the distributions of predictor variables for normality using standardized normal probability and kernel density plots (pnorm, qnorm, and kdensity commands). Those with non-normal distributions were considered for transformation prior to multivariate analysis using linear regression modeling in order to meet the assumptions of the model. All data were analyzed using Stata 12 (StataCorp, College Station, TX).

We compared the resulting penetrance heat map to known geographic, political, and sociodemographic maps of the state. A qualitative comparison of penetrance “hot spots” (areas of increased penetrance) and “cold spots” (areas of decreased penetrance) to areas of known sociodemographic or geographic importance was then made. The assessment of importance was made by PC staff, medical toxicologists and medical toxicology fellows, based on PC-identified areas of interest. As discussion of heat mapping of each of more than 1300 USCB tracts was infeasible for the purpose of a single study, we highlighted previously unidentified geospatial findings of potential clinical importance to the PC as exemplars of the utility of geospatial analysis for PCs.

## RESULTS

**Figure 1:**
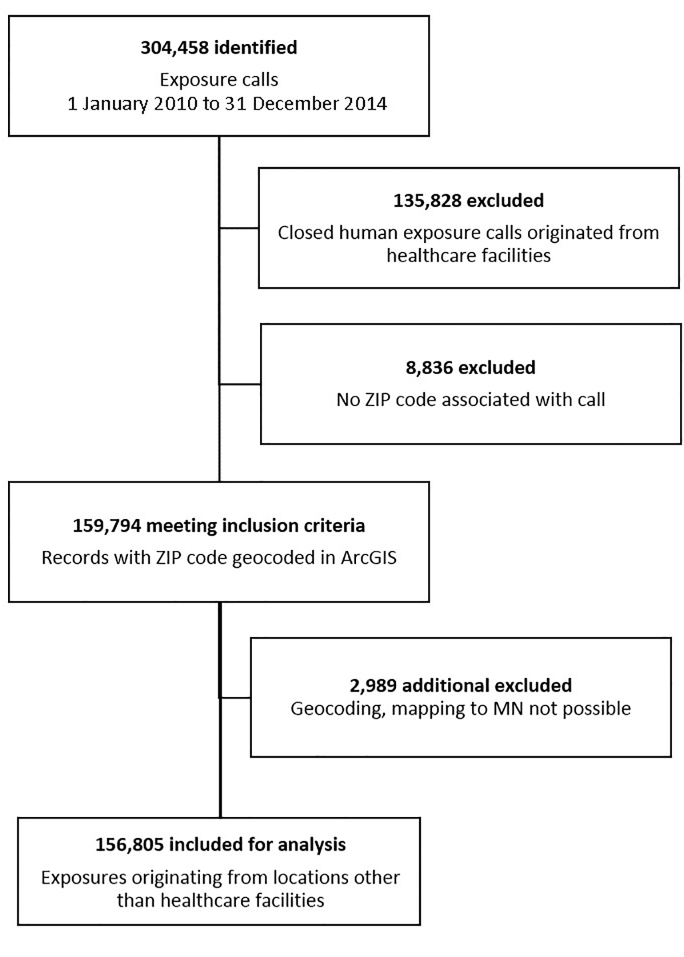
Study flow diagram of phone calls made to Minnesota Poison Control System, using geospatial analysis to pinpoint origin.

Annual call volume to the PC ranged from approximately 51,000 to 58,000 calls during the study period; of these, approximately 85–89% were exposure calls annually, and 77–81% were unintentional. Over the five-year study period, 304,458 exposure calls to the PC were identified (Figure 1). Of these we excluded 147,653, largely accounted for by those originating from healthcare facilities (91.99%). Smaller exclusions were due to missing ZIP Codes (5.98%) or ZIP Codes that were not mappable to the state (2.02%). The remaining 156,805 exposure calls not originating from healthcare facilities were included for regression analysis and geospatial mapping.

Non-normal distributions of observations were noted for all variables but the proportion of population less than five years of age. Numerical and graphical evaluation suggested square root variable transformations as most appropriate to meet the regression assumption of normally distributed data for all but the population proportion reporting educational attainment of eighth-grade or better to the USCB. In that case, transformation did not meaningfully impact observation distribution, and was not applied. Post-hoc model assessment revealed heteroskedastic distribution of regression residuals (estat hettest, Breusch-Pagan χ^2^ 303.6, p = 0.000), and thus robust standard errors were applied to the model.

Linear regression revealed significant associations between PC penetrance and USCB tracts with higher proportions of eighth-grade educational attainment or higher (*β* = 5.05, p = 0.008), non-Hispanic Blacks ((*β* = 1.18, p = 0.032), and American Indians ((*β* = 3.10, p = 0.000), indicating that census tracts with higher proportions of these demographic groups would be expected to display greater PC penetrance. No significant association was noted between PC penetrance and population proportions below the federal poverty line, proportions identifying as Asian, Hispanic, non-English speaking, or proportions of population less than five years of age. Variance in penetrance explained by regression modelling was low (R^2^ = 0.054).

**Figure 2:**
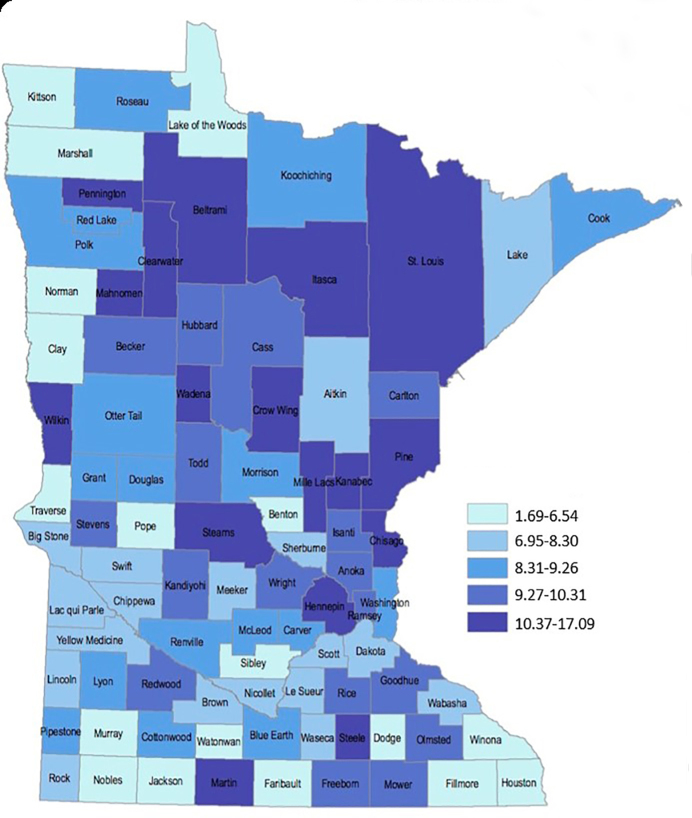

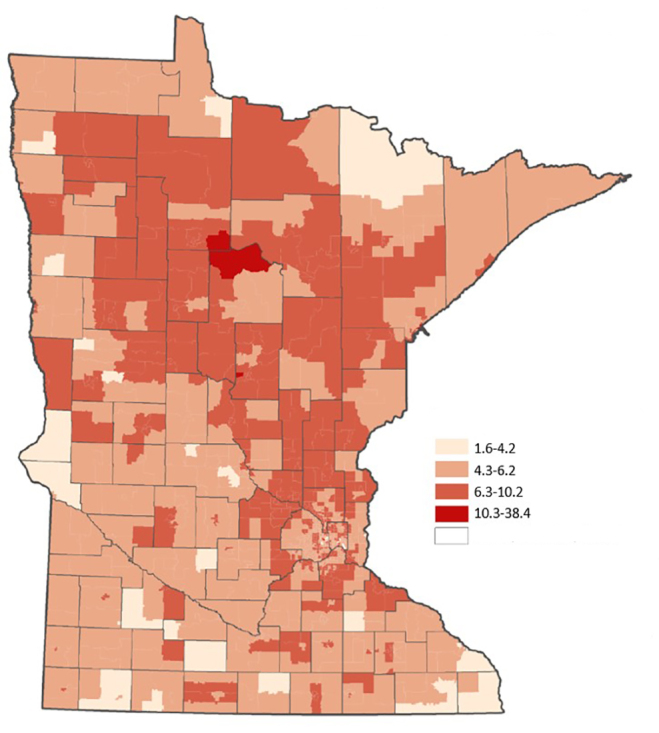
A) 2012 distribution of poison center penetrance (calls per 1000 population) prior to geospatial mapping of all calls. Legend reports penetrance as calls per 1000 residents per year. B) 2010 – 2014 census tract geospatial mapping of poison control call penetrance. Legend reports penetrance as calls per 1000 residents per year over the study period.

Previous county-based geospatial penetrance mapping (Figure 2b) revealed a caller distribution profoundly more complex than previously available county-wide penetrance maps (Figure 2a), with substantial intra-county variability in PC penetrance. “Cold spots,” or regions of low penetrance, were identified in southern, southeastern, and west central regions of the state, while “hot spots,” or regions of increased penetrance, were identified in small north central and northern areas of the state, and within the state’s two largest urban centers. These consequential variations in the geospatial distribution of PC calls were not captured by statistical modelling. Case examples elucidate nuances to call distribution not captured by regression analysis.

### Case Examples

#### Leech Lake Reservation

**Figure 3:**
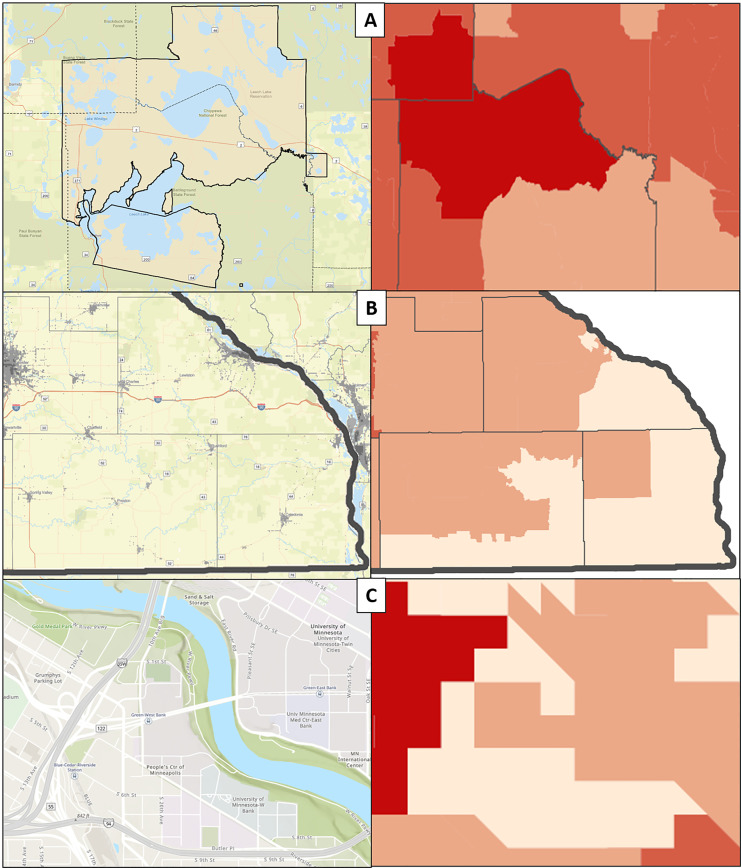
Case examples. A) High penetrace region at the confluence of three rural counties and overlying Leech Lake Reservation. B) Low penetrance region in far southeastern Minnesota. C) Low penetrance region correlating with the Cedar-Riverside neighborhood of Minneapolis.

An isolated penetrance “hot spot” in north central Minnesota correlated with the intersection of Cass, Beltrami, and Itasca counties (Figure 3a). No regional suggestion of increased calls was apparent by county-based spatial mapping of call penetrance (Figure 2a). Census-tract spatial distribution of penetrance revealed a hot spot substantially and uniquely overlapping the legally designated Leech Lake Indian Reservation. This finding suggests a previously undetected variation in penetrance within the reservation with no clearly apparent etiology.

#### Southeast Minnesota

A “cold spot” was identified in far southeastern Minnesota correlating with Fillmore, Houston, and Winona counties (Figure 3b). All three counties were low penetrance by county-based mapping; however, census tract mapping revealed that the extreme southeastern component of the area had considerably lower penetrance than the northern and western portions of the counties. This subregion represents the most sparsely populated area of the three low-penetrance counties, and correlates with one of the 25 largest Amish settlements in the US as a percentage of county population (4.69% of Fillmore county in 2010).^29^ Amish communities commonly de-emphasize ownership or use of private telephones,^30^,^31^ and PC penetrance within this community may thus be constrained by technology, suggesting a need for further exploration of this finding, and for consideration of alternate communication methods in areas of low telephone availability.

#### Cedar-Riverside

A “cold spot” was identified in central Minneapolis overlapping Cedar-Riverside (Figure 3c), a triangular neighborhood contained on two sides by freeways, and on the other by the Mississippi River. Forty-eight percent of the Cedar-Riverside population is Black, while 51.3% of the population there speaks a language other than English.^32^ This diverse neighborhood is the epicenter of Minnesota’s Somali diaspora, estimated between 27,000 born in Somalia and 46,000 reporting Somali ancestry.^33^ The western and southern regions of Cedar-Riverside are more heavily populated by the Somali population, while the northern and eastern regions are occupied by the University of Minnesota campus. Low PC penetrance appears limited to areas of Cedar-Riverside with the highest Somali population density, while the remaining neighborhood heat map displays no observable low penetrance.

## DISCUSSION

In a regional PC in a state with significant racial and cultural disparities, USCB-defined characteristics of greater than eighth-grade educational attainment, non-Hispanic Black identity, and Non-Hispanic American Indian identity were associated with increased call penetrance to a PC. This suggests increased PC utilization among those with higher educational attainment and those who identify as Black or American Indian. Our findings share some of the findings reported in 2010 by Litovitz et al, who noted an increase in penetrance in populations with high percentages of residents with Asian background, residents younger than five years of age, and residents holding bachelor’s degrees, among others.^5^ Our studies stand in distinction to the findings reported by Vassilev et al, who identified high population density and high proportions of non-White races as predictors of low, rather than high, PC utilization.^34^ Still other studies have identified Hispanic background as a negative predictor of PC utilization^12^; our results describing this association did not achieve statistical significance, despite suggesting a similar relationship.

Nonetheless, in the context of finite PC resources, the results of regression modeling are useful but insufficient to plan strategic and cost-effective PC outreach. Regression modeling alone cannot identify specific geographic regions of low penetrance, and ultimately this is inadequate to implement fully informed, ground-level decisions regarding resource utilization and geographic targeting of PC outreach. Routine statistical modeling, therefore, provides a conceptual framework for understanding PC penetrance, while geospatial mapping offers a direct assessment of low and high penetrance areas of interest on which PCs may focus outreach resources.

The three cases of Leech Lake, southeast Minnesota, and the Cedar-Riverside neighborhood of Minneapolis provide unique examples of regions inadequately described by statistical modeling and prior county-level geospatial descriptions of PC penetrance. The etiology of increased PC penetrance in the Leech Lake region is obscure but consistent with regression modeling, and this “hot spot” was not identified prior to granular PC penetrance mapping. While a culture of increased utilization may exist across residents of this geographic region, a single “super user” in a sparsely populated region may also be responsible for this finding. Alternately, a higher than expected volume of exposures reported from non-healthcare locations may be related, warranting further public health outreach and PC investigation. Finally, in a resource-poor area of the state, access to expert medical opinion regarding poisonings may be more feasible by phone than by physical presentation to a medical provider.

In southeast Minnesota, multiple plausible explanations for decreased penetrance exist. A relatively large proportion of the regional population is of the Amish faith, and many are likely without telephone service in their homes. While other regions of Minnesota are home to significant Amish populations, few are as large or established, and most are much more recently founded. This raises the possibility of important cultural differences, including telephone ownership, between older and more conservative Amish communities in southeast Minnesota and more recently founded, more progressive communities in other regions.^29^ Despite prior studies identifying mass-mailing campaigns as ineffective in reaching rural populations^35^ and increased rural call volumes following the implementation of toll-free access to PCs,^36^ this region may stand in contradistinction given the higher than normal proportion of residents with minimal access to technology including telephones and electricity. Lastly, our findings may simply identify an area where PC outreach efforts have heretofore been inadequate, where lower than expected rates of poisonings occur, or where poisoned patients and those around them more commonly present to healthcare facilities than contact the PC.

Finally, Cedar-Riverside represents an area of particular concern for the PC, and likely reflects challenges experienced by other PCs. While the volume of PC calls using a telephonic language-interpreting line remained very low as a percentage of all calls over the study period, no prior efforts had been made to objectively study our poor penetrance into language minority groups. The present study strongly suggests that the PC is not attending to one of the largest regional minority groups. During the study period, Somali language interpreters were used for only four calls, and as recently as 2015–2017, Somali interpreters were used for 3–5 calls annually despite a known population of more than 40,000. Whether this poor penetrance represents sociocultural or linguistic barriers, low awareness of PC services, or a low rate of poisonings in this subgroup is unclear, and suggests an avenue to which outreach resources may be directed.

## LIMITATIONS

Several limitations govern the interpretation of these findings. This cross-sectional study in a single state identifies associations between PC penetrance and USCB-defined variables, but causal relationships between demographic variables and penetrance variation cannot be inferred. Generalizability to other PC catchment areas is not described. Similarly, a high risk of type I statistical error is inherent to large datasets such as this: many UCSB component variables are available for statistical modeling, raising the risk of inappropriately focusing on unexpected associations or findings. To mitigate this, we identified variables of interest a priori, and did not add to our model thereafter. While our resulting regression model explained little of the variability seen in our study, this was likely a result of confounding by multiple factors, one of which is the geographic distribution of callers that we sought to study through geospatial mapping. Indeed, the limited utility of statistical modeling, absent geospatial mapping, is an important and central finding of this study.

Additionally, the assessment of penetrance in this study is rooted in its historical utilization both as a marker of PC efficacy and for accreditation through the AAPCC. The use of penetrance as an accreditation metric was discontinued in 2001 absent data to support its use. However, data from this era are characterized largely by evaluations of penetrance as it relates to differences in populations’ ages, specifically the proportion of the population younger than two years old, at a time when counties were largely considered the unit of measurement, and when further geographic subanalyses would have been less accessible. Penetrance, described at a much more granular level of analysis, better defines areas of low PC utilization, inviting further evaluation prior to the redistribution of PC resources and suggesting that penetrance may yet hold value for PCs.

An additional limitation of our dataset is the predefined nature of USCB data. Within USCB-defined variables such as “non-Hispanic Black,” more nuanced associations, unique to our state, may exist between PC penetrance and subgroups otherwise subsumed under USCB variables (for example, both Karen and Hmong cultural groups coding to “non-Hispanic Asian”). This limitation is at the root of the present study, which seeks to better identify underserved groups through geospatial mapping.

We excluded calls coded as originating from healthcare facilities, but miscoded or misreported calls may have been inadvertently included in the study. Nonetheless, a small number of miscoded cases is likely mitigated by the overall large number of observations. Similarly, callers from mobile phones with area codes mapping to Minnesota may have called the PC from outside the state, causing inclusion of calls from an unintended region. Callers from mobile phones with area codes mapping outside of Minnesota, but residing within the state, may have been inadvertently excluded. This is likely addressed, however, by exclusion of such calls when documenting caller-reported ZIP Codes not mapping to Minnesota at call initiation.

Finally, spatial apportionment of US ZIP Codes to USCB tracts is a good measure of population parameters, but it imparts a small degree of imprecision when combining these datasets, both of which are characterized by similar but unique geographic boundaries. In describing penetrance, this imprecision, likely to occur on the edges of identified boundaries, is unlikely to meaningfully affect the interpretation of results intended to geographically guide outreach efforts. While some case records report addresses, far more contained ZIP Codes, making this a more adequate data point to map calls. Further, the extraction of addresses was not feasible due to limitations in data extraction from local call management software. Additionally, ZIP Codes may change periodically, but it was beyond the scope of this investigation to identify small changes to ZIP Code areas, potentially imparting further imprecision to our findings.

## CONCLUSION

In this investigation, historically employed statistical and county-based methods to define poison center penetrance fail to recognize systematic failures to reach specific demographic and geospatially defined groups. Higher American Indian and non-Hispanic Black population proportions, and greater than eighth-grade educational attainment, are characteristics associated with increased PC penetrance in this study. Evaluating the geospatial distribution of calls to other PCs may enhance understanding of penetrance patterns, improve resource allocation and elucidate previously unknown predictors of PC penetrance. This novel and detailed visual account of PC penetrance, uniquely interpretable when contextualized in a knowledge of the state served by the poison center, offers a new tool to optimize PC outreach.
